# Feasibility study of a portable transparent vinyl chloride shield for use in an ambulance during the COVID-19 pandemic

**DOI:** 10.1186/s13054-020-03381-9

**Published:** 2020-11-19

**Authors:** Kohei Tsukahara, Hiromichi Naito, Tsuyoshi Nojima, Takashi Yorifuji, Atsunori Nakao

**Affiliations:** 1 Department of Emergency, Critical Care and Disaster Medicine, Okayama University Graduate School of Medicine, Dentistry and Pharmaceutical Sciences, 2-5-1 Shikata-cho, Kita-ku, Okayama-shi, Okayama, 700-8558 Japan; 2 Department of Epidemiology, Okayama University Graduate School of Medicine, Dentistry and Pharmaceutical Sciences, Okayama, Japan

## To the Editor,

An emergency medical technician (EMT) is frequently the first healthcare provider that COVID-19-positive patient encounters, and faces significant risk during procedures with the potential for aerosolization including advanced airway management and cardiopulmonary resuscitation. Polycarbonate devices for shielding droplet splash and aerosols have been adopted by some hospitals [1]. However, placing heavy sizable barriers in an ambulance increases the risk of injury to both the patient and EMT during airway management and may pose kinesthetic challenges and increase time to intubation [2, 3]. Reducing these risks was our highest priority in designing a portable shield for ambulatory care.

**Fig. 1:**
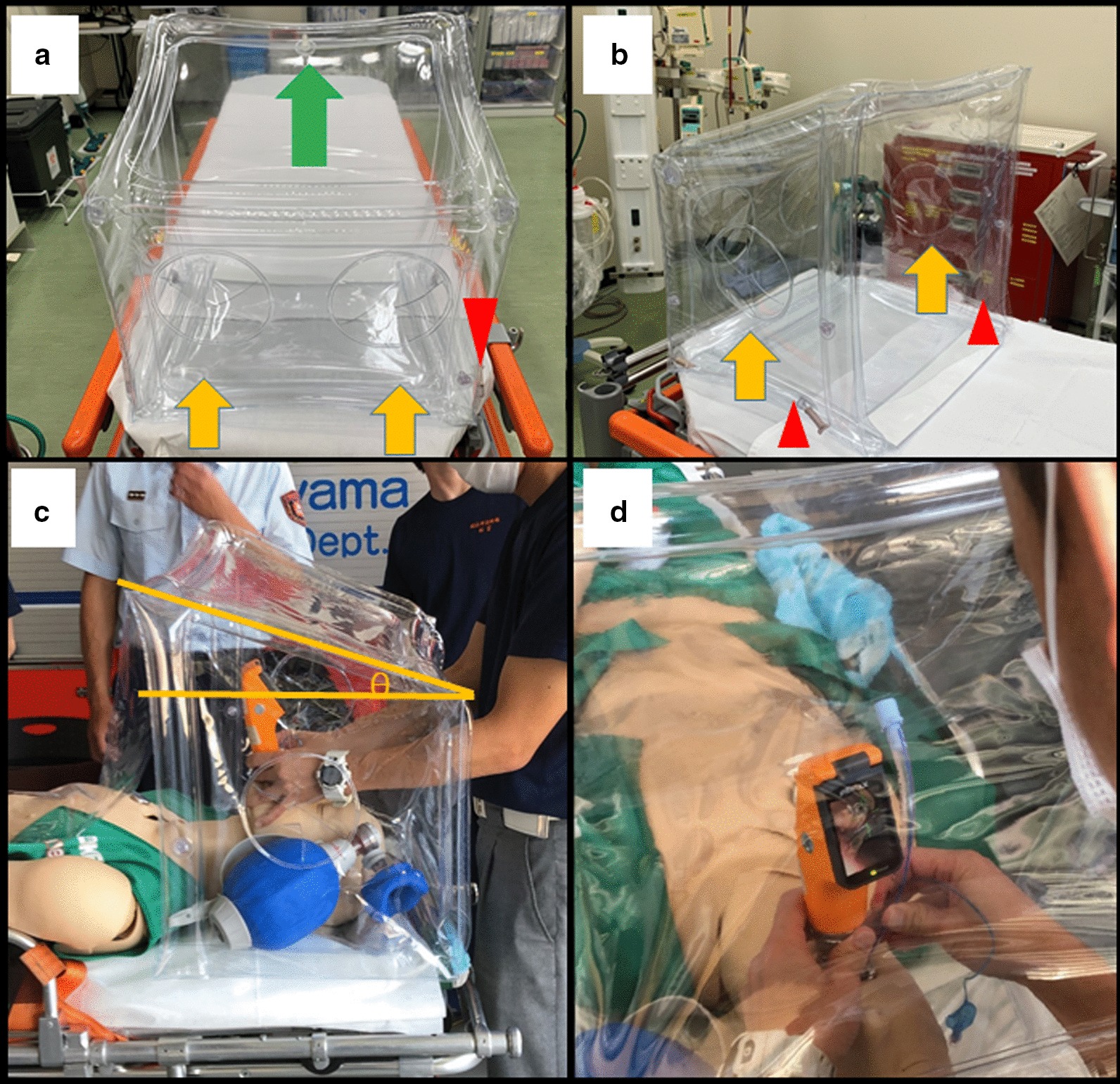
Portable aerosol shield. The shield is made of transparent vinyl chloride and can be set on the stretcher during patient transportation. It has four arm ports (radius 150 mm, indicated by yellow arrows), two in the front (**a**) and one on each side (**b**), one suction port (green arrow) and six injection/oxygen ports (red arrowheads). The top of the shield is sloped ~ 20° to increase visibility (**c**). Tracheal intubation was performed with the portable shield in place using video-laryngoscope (AWS-S100, Nihon Kohden, Tokyo, Japan) (**c**, **d**), and the view from the EMT’s perspective is shown (**d**)

**Fig. 2:**
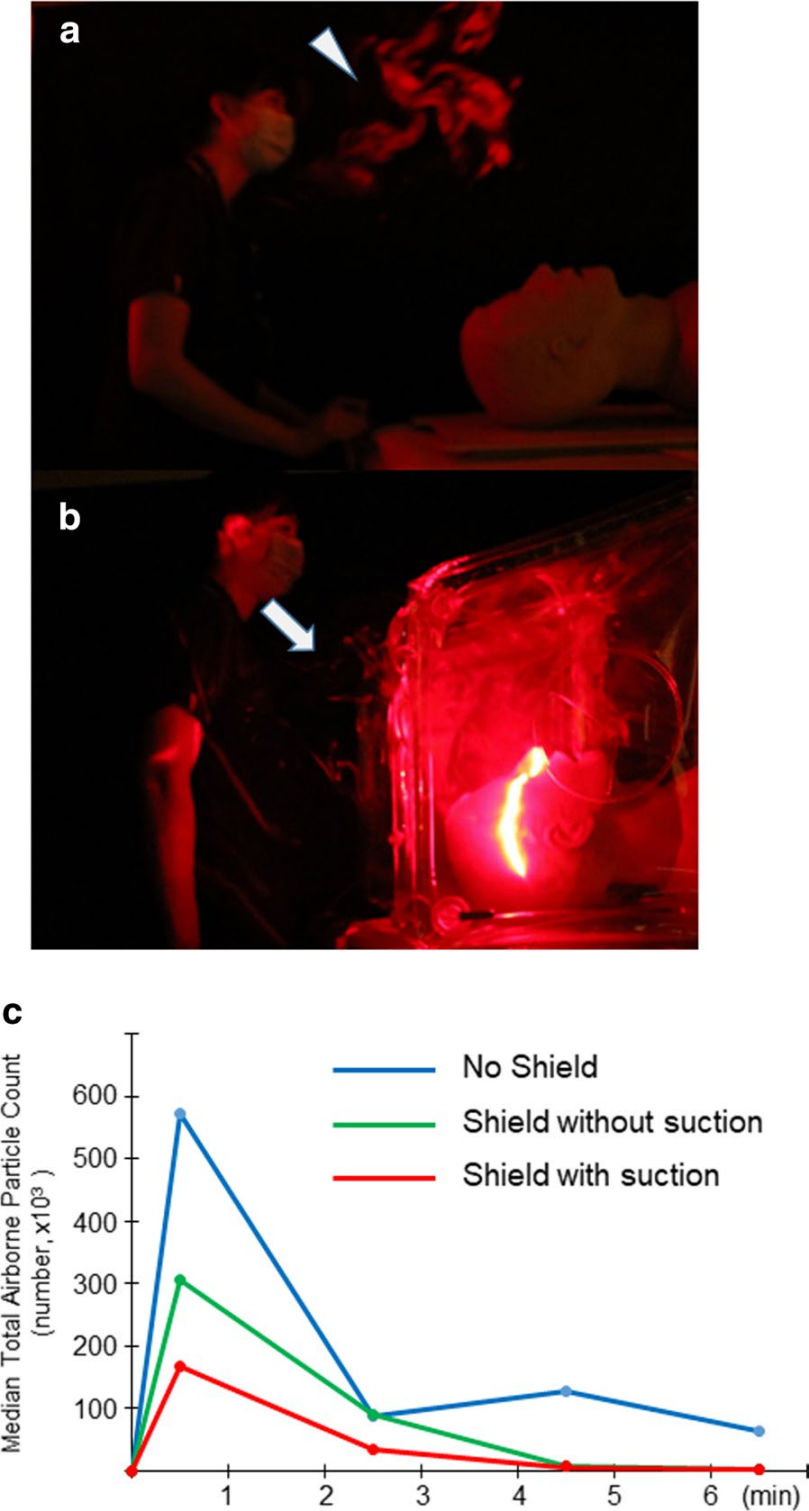
Visualization of an aerosol with and without the portable shield. Aerosol was generated using an Atomizer Aerosol Generator ATM226 (TOPAS, Dresden, Germany), dispersed through a tube placed in the manikin’s mouth, and visualized with laser-light scattering. Without the shield, the aerosol reached to the operator within 30 s (white arrowhead, 30-s time point is shown) (**a**). The portable shield drastically reduced exposure of the operator to the aerosol (white arrow, 30-s time point is shown) (**b**). Particle counts 50 cm away from the mouth of the manikin using a Kanomax Portable Particle Counter (Model 3889, EndoKagaku, Shizuoka, Japan) revealed that the shield effectively minimized aerosol dispersion when suction was applied to create negative pressure (**c**)

The portable shield was fabricated with transparent vinyl chloride in cooperation with HibiiX Co, Ltd (Mizuho, Japan), a company that produces swim floats. The device has a relatively sturdy frame and automatically inflates by lifting the frame upwards. The inflated shield is 50 × 50 × 40 cm and weighs 850 g; the deflated shield is 25 × 20 × 5 cm. There are four ports for the EMTs’ hands, one suction port, six injection/oxygen ports, and a flap on one side (Fig. 1). The device is reusable after disinfection with hypochlorite and ethanol. Recently, the United States Food and Drug Administration recommended that healthcare providers should not use passive protective barrier enclosures without negative pressure, as they may not decrease exposure to airborne particles, and in some circumstances, may increase exposure [4]. Therefore, continuous suction can be applied to maintain negative pressure inside of the shield while in use. Laser-flow visualization demonstrated that with suction to generate negative pressure, the shield reduced aerosol dispersion and exposure to airborne particles (Fig. 2) [5].

Ten different right-handed EMTs tested the device during a routine training course after providing written consent to participate. Ethics Committee approval was obtained (K2010-008). The participants had worked for a median 10.2 years (range 7–14 years) as tracheal intubation-certified EMTs. During the training session, the EMTs received 30 min of oral instruction on tracheal intubation with video laryngoscopy, insertion of a laryngeal tube (LT), and manual ventilation using a bag-valve mask (BVM). Each EMT then performed ten intubation trials on an adult-sized manikin: five without the shield and five with the shield. Participants were timed and ranked the feasibility of the using the shield on a scale of 1–10. A score of 5 indicated an equal experience to performing the procedure without a shield; 1 was the lowest possible score.

The intubation success rate was 100% for all trials. The average intubation time under the shield was 15.38 ± 11.9 s as compared with 12.6 ± 9.0 s without the shield (*p* = 0.24; *T* test analysis). When the feasibility of using the shield was assessed by EMTs, the feasibility scores were 3.6 ± 0.7 for intubation with video laryngoscopy, 3.1 ± 0.7 for insertion of a LT, and 4.2 ± 0.8 for ventilation using a BVM. Finally, we confirmed in patients that the shield did not interfere with BVM ventilation in the ambulance (*n* = 10) or endotracheal intubation in the emergency department (*n* = 2).

These data indicate that this lightweight, easy-to-store, vinyl chloride shield is a feasible tool to securely cover the face of a patient during transport and reduces viral exposure, although some aerosol leak may still occur. The shield allowed adequate visualization without loss of function and may reduce the high risk of viral contamination imparted by aerosol-generating procedures during emergency medical transport. The shield design is supported by a recent proof-of-concept study demonstrating that plastic drapes significantly limit aerosolization and droplet spray [6]. Intubation devices should be prepared inside the shield before the suction tube is placed through the side port. Minimizing viral transmission during transport is essential as the world navigates the COVID-19 pandemic.

## Data Availability

The data are available from the corresponding author, HN, upon reasonable request.

## Abbreviations

BVM: Bag-valve mask
COVID-19: Coronavirus disease 2019
EMT: Emergency medical technician
LT: Laryngeal tube
